# From Folklore to Scientific Evidence: Breast-Feeding and Wet-Nursing in Islam and the Case of Non-Puerperal Lactation

**Published:** 2007-12

**Authors:** Lia Moran, Jacob Gilad

**Affiliations:** 1 *The Centre for the Study of European Politics and Society, Ben-Gurion University of the Negev, Beer-Sheva, Israel;*; 2 *Clinical Microbiology Laboratory, Tel-Aviv Sourasky Medical Center, Tel-Aviv, Israel*

**Keywords:** breast-feeding, lactation, muslim, puerperium, folklore, wet-nursing

## Abstract

Breast-feeding practice has an important medical and socio-cultural role. It has many anthropological aspects concerning the “power structures” that find their expression in breast-feeding and the practices that formed around it, both socially, scientifically, and legally-speaking. Breast-feeding has been given much attention by religions and taboos, folklore, and misconception abound around it making it a topic of genuine curiosity. This paper aims at expanding the spectrum of folklore associated with breast-feeding. The paper deals with historical, religious, and folkloristic aspects of breast-feeding, especially wet-nursing, in Islam and focuses on an intriguing Islamic tale on breast-feeding - lactation by non-pregnant women (or non-puerperal lactation). Apparently, accounts of non-puerperal lactation are not restricted to Islam but have been documented in various societies and religions throughout centuries. Two medical situations - hyperprolactinemia and induced lactation, appear as possible explanations for this phenomenon. This serves as an excellent example for the value of utilizing contemporary scientific knowledge in order to elucidate the origin, anthropology and evolvement of ancient myth and superstition.

## INTRODUCTION

Western societies are preoccupied with the sexual-esthetic function of the female breast. However, its true wonder lies in the power to lactate, the maternal attribute that has enabled mammals to survive over millenia. Breast-feeding practices have an important medical and socio-cultural role. Lactation has a direct beneficial effect on the infant in that it promotes its growth and normal development and confers protection against various infantile diseases, especially infections. In addition, lactation is far more than merely a biological fact: it is an aspect of “mothering”, the culturally constructed bonding between a mother and her child (1).

The frequency, duration, and methods of breast-feeding are influenced by many socio-cultural, geographic and economical factors and have been subject to various changes across centuries. Moreover, it has many anthropological aspects concerning the “power structures” that find their expression in breast-feeding and the practices that formed around it, both socially, scientifically, and legally-speaking.

Breast-feeding has also been given much attention by religions and has always fascinated mankind. Myths, superstitions, taboos, folklore, and misconception abound around it making it a topic of genuine curiosity. This paper, aims at expanding the spectrum of folklore associated with breast-feeding. The paper focuses on historical, religious, and folkloristic aspects of breast-feeding, especially wet-nursing, in Islam and deals with an Islamic tale on breast-feeding - lactation by non-pregnant women - with an attempt to provide scientific explanations for this lore.

## BREAST-FEEDING AND WET-NURSING IN ISLAM

Wet-nursing, or the breast-feeding of another woman’s child, either in charity or for payment, occurred in all civilizations in which the death of the mothers in childbed or during lactation was relatively common, but this was not the only reason why it was employed. Social, political and religious factors played an important role in determining the incidence and extent of professional breast-feeding in different societies throughout history. In some civilizations wet-nursing occurred mainly on a casual basis: where lactating relatives or neighbors fed another child along with (tandem lactation), or after weaning their own infant. In others, it was a highly organized practice among certain classes of the population, and wet-nurses and parents were subject to the law of the land or to that of their religion (2).

Islamic law defines three types of kinship: relationship by blood, marriage and milk. The last is referred to as *al-rida’a* in Arabic. All three involve an impediment to marriage between certain persons so related. The milk relationship is a restricted form of legally recognized kinship: milk kin cannot be inherited from each other; milk parents have no legal duty to maintain their milk children; nor do they have any form of guardianship over them. Milk relationship comes into existence through a woman breast-feeding another’s child. Although for all practical purposes the milk-mother herself fulfils the same role as the wet-nurse in former times in Europe, it differs basically from that institution since the latter did not involve the child and the nurse in any legally recognized relationship (3).

The special term *rida’a*, denotes the relationship between a child and a woman, not his own mother, who nursed him (4). The rules defining the relatives of a person whom he or she may not marry are straightforward for consanguineal and affinal relatives. In this case, the prohibition derives from the doctrine that the “fluids” of both the lactating woman and her husband generate the milk. The doctrine refers not to the woman’s husband but her mate (“copulation partner”). Consequently, a boy cannot be the milk-son of a man who either married the boy’s milk-mother after she nursed him or divorced her. In establishing the range of forbidden marriages, a child nursed by a woman is treated as if he was the child of her husband, so that two children nursed by the same woman are regarded as if their milk-mother’s husband were their common milk-father even if both children have different parents. It follows that a boy and a girl each nursed by a different wife of the same man become his milk-children and milk-siblings to each other (5).

It should be clear by now that in many societies the rules regarding breast-feeding, were laid down by men, and tend to support male-dominated institutions. For example, in those countries which observe Muslim civil law, the duty of a woman to feed her husband’s children, the duration of feeding and the conditions under which she may feed children other than her own, thus establishing links of milk-kinship, are all prescribed by a male-dominated paternal legal system. The feeding of one woman’s child by another has been used in different societies to make peace between two tribes, to consolidate clan unity, to prevent marriage, to create clients, and in sum, to attain objectives which lie far beyond the nursing woman’s own interest.

In many societies we may find a “muted” or “counterpart model” which stresses women’s reproductive power and their intrinsic physical benevolence. Yet in these same societies, a woman’s milk is a sign of the blessing and abundance (*baraka*) that she brings to her husband’s household, fields, animals, and on which his prosperity depends. That is, breast milk is a female source, whose cultural and institutional importance is such that men and women contend for its control. The mode and circumstances of breast-feeding are also considered in many societies to be fundamental in the definition of mother-child and in general of adult-child relationship.

Muslim societies commonly oblige a woman to breast-feed her husband’s children for two years (6). Nevertheless, the Koranic recommendation is less stringent than that of many contemporary Muslim societies (7). The importance of breast-feeding in both the Islamic religion and society cannot thus be overstressed and accounts for the substantial proliferation of breast-feeding folklore in the Islam.

## BREAST-FEEDING IN ISLAMIC FOLKLORE

A rich folklore with regard to breast-feeding has emerged during the ancient times within various cultures and religions. Specifically, Islamic societies relied on many common beliefs and superstitions, some of which were based on the writings of Islamic doctors, e.g. that milk was claimed to transmit mental and moral traits to the infant in addition to its presumed physical benefit. Other myths have sometimes been developed with the aim of achieving social, religious, or cultural goals, such as tribe expansion under milk kinship laws. Another example is the Islamic concept that sexual intercourse with a nursing woman is harmful, both for the nursling and the woman herself if she is carrying a fetus. This myth has been used in some paternal societies as a justification for handing over newborns to wet-nurses so that men may resume sexual activity soon after their wives have given birth (8).

There is no private domestic sphere, where it is more proper to breast-feed than at work. In sexually segregated societies such as those of North Africa and the Middle East, women breast-feed in the company of other women, who are not always kin. Among the *`awa`il* of the Hejaz (Saudi Arabia), elderly women often talk about how an envious woman can, just by looking at another nursing, cause the latter’s milk to stop or make her ill. To feed only in the presence of family is only one of many precautions which Hejazi women adopted to avoid the “suppression” of milk (9).

In the Northwestern Mountains of Tunisia, the regions which French colonists called Khroumirie, where the Khmir people live, women have additional reasons for appreciating breast-feeding. There, it is a cultural axiom that the flowing of a mother’s milk and her baby’s feeding on it is a sign of *baraka,* a life sustaining force. When a mother transmits this force, it is not only the nursling who prospers, but also everybody and everything pertaining to the house. Hence people feel confidence in their general circumstances when a baby fares well.

The best time to study ideas of nursing is when the baby falls ill. The Khmir think of a baby as a creature perfect by nature. In practice this means that when a baby falls ill, the explanation that is given most frequently is that his mother’s milk did not suit him - *hilb msuma* (“Bad Milk”). Bad Milk may occur at any stage of the lactation period, with the exception of the first forty days. It is a symbolic number, standing for the fulfillment of motherhood and the perfect condition of the mother-child unity.

It is believed that there are five variants of children’s illness, caused by Bad Milk: *bunaghut, afn, shedd, felg* and *helg.* They have no generic name and the differences between the variants are of lesser importance. A general opinion in the villages is that *bunaghut* is caused by the baby’s feeding on the breast just after the mother had come back from fetching wood in the mountains. People believe that the mother’s milk becomes worm and churned up because of the overheated condition of the whole body. The milk in the breasts presumably returns to its normal state after half an hour. Supernatural agents have never been implicated in the change in the milk. Milk illness is cured by carrying out a ritual that affirms that mother is indeed able to spread *baraka.* After the treatment the baby accepts the breast again and the symptoms of illness disappear (8).

## GENERAL FOLKLORE OF BREAST-FEEDING

The Roman physician Soranus advised on hiring a wet-nurse in as early as the first century A.D. (9). It was said that the best wet-nurse should be chosen. The preferred profile of a wet-nurse was a woman aged 20 to 40 years (experienced but not too old), that had given birth twice or thrice and has been nursing for at least three months, healthy and of large frame (thought to be more nourishing) with medium-size unwrinkled breasts and nipples, that does not drink (which may be harmful to the nursling), and is not ill-tempered (as character was thought to pass with milk).

The ‘Evil Eye’ is the name for a sickness transmitted, usually without intention, by someone who is envious. The evil eye belief existed throughout centuries among various geographic areas and religions. Basically, the evil eye is based upon an underlying belief about water equating to life and dryness to death. Specifically, the true “evil” done by the evil eye is that it causes living things to “dry up” (10). In the context of breast-feeding and infant nursing, the evil eye has been mostly implicated in the overlooking, projection, or praising by envious women, childless women, or those with blue eyes, that resulted in the drying up of the mother’s milk or illness and crying of the nursling (the same could have happened to nursing cows or goats). The evil eye could be prevented or cured by numerous methods, including spitting on the child, amulets, horseshoes, and wax dripping (10).

The ability to stimulate or suppress milk production has been attributed to many folklore herbs and foods. Alfalfa, peanuts, beans, coffee, anise, and fennel have all been related with increased milk production (11). Sesame-seed cakes are still being used in Mexico while Goat’s milk is used in India. Other folkloristic methods include relaxation with soothing music that was popular during the Renaissance period or using plant poultices covered with heated stones.

Many old myths regarding the process of breast-feeding can still be encountered today despite the vast scientific progress and are attributed mainly to ignorance. Some examples recently addressed in the midwifery literature are a connection between yellow colostrum and neonatal jaundice, that the breasts empty after a feed, that there is less milk in the evening, that a mother should drink and eat a lot to make milk, that drinking cow’s milk makes human milk, and finally that small breasts do not make enough milk (12). With regard to the latter, the Aboriginal Juangs of India actually consider small flat breasts as the most effective milk producers (11).

## BREAST-FEEDING FOLKLORE IN CHRISTIANITY AND JUDAISM

In the medieval period, the female breast was regarded as a sacred object and breast-feeding was assigned many religious roles apart from maternal imagery. Breast milk at these times was believed to be menstrual blood that had been heated, coagulated, and whitened by hot air according to the Jewish Talmud, Aristotle, Galen, and later Middle-Age philosophers. The concepts of breast-feeding in Christianity have been implicated mostly with regard to Virgin Mary and her child Jesus Christ.

Contrary to modern days, Jesus Christ has been often portrayed as having feminine qualities in medieval times (13). This includes both having physical feminine attributes such as lactating breasts as well as religious ones, such as Christ lactating his believers, reversing the role of Mary and Christ-child to Mother Jesus and the child-like soul. Others have connected the wound in Jesus’ side and breasts full of soul-sustaining milk or used breast milk symbolism to illustrate ideas of the motherhood of Christ versus the fatherhood of God (14).

The breasts of Virgin Mary have probably been depicted in more images than the breasts of any other woman in history. Mary’s breasts are full of not only milk but fraught with symbolism and spirituality. The Virgin and Christ are commonly depicted in the intimate embrace of breast-feeding (designated ‘Maria Lactans’) which signifies the humanity of Christ and the gift of God and occasionally grace and humility (15). It has been speculated that the ‘Maria Lactans’ had originated from the pagan Egyptian descriptions of the goddess Isis nursing her son Horus.

The Old Testament and various Jewish religious sources also refer to breast-feeding on many occasions. The first reference in Hebrew Scriptures is found in the book of Genesis (21:17). One of the most famous stories on wet-nursing appears in Exodus where Pharaoh’s daughter sends Miriam to call for a wet-nurse for baby Moses (Exodus 2:7-10). Similar with the Koran, many recommendations with regard to the preferred timing for weaning are made in the Bible. As mentioned with regard to Christianity, breast-feeding was also used metaphorically, e.g. the city of Jerusalem pictured as a nursing mother to her inhabitants (Isaiah 66:10-12). Moreover, successful breast-feeding has been brought as a benediction (Genesis, 49:25) while dry breasts were the symbol of a malediction (Hosea, 9:14).

## NON-PUERPERAL LACTATION

As described above, a rich folklore has emerged within Islamic societies with regard to breast-feeding. One such lore is wet-nursing by women who have never given birth (nulliparous women) or who have never been pregnant (nulligravid women). Specifically, the Bedouin tribes of the Negev area in Southern Israel tell the tale of a woman who has been unable to give birth. When her husband consequently married a second woman, she miraculously developed the ability to lactate the second woman’s new-born. Whereas professional nursing usually represents the physiological continuum of a woman’s ability to breast-feed other infants after giving birth to a child of her own, breast-feeding that does not follow a normal post-partum period is rather enigmatic and intriguing. Is this phenomenon actually feasible? Can the origin of this lore be elucidated by contemplating scientific evidence?

Breast-feeding following birth requires a series of physiological processes that begin during pregnancy itself and continue post-partum. For lactation to take place, both structural changes within the breast tissues and hormonal regulation are mandatory, especially estrogen-mediated prolactin (PRL) secretion (16). PRL secretion increases with both psychological and physical stimuli such as suckling or breast manipulation. Normally, without suckling, milk production ceases 14 to 21 days after birth. PRL- mediated milk production and secretion, however, may continue as long as the breasts are stimulated, as evidenced by the ability of wet-nursing for many years (16).

### Earlier Accounts of Non-Puerperal Lactation

Non-puerperal lactation has been described in the literature on several occasions. The ascetic mystic Christina Mirabilis had two miraculous experiences with relation to her breasts, as suggested by her biographer Thomas de Cantimpre. In the first occasion, Christina escaped torture after believed to be possessed by her town people. After pleading mercy to her God, her virginal breasts were dripping sweet milk so she could feed herself for nine weeks while hiding. Christina was portrayed similar to the incomparable Mother of God (17). However, although both were known as virgins, Mary had been pregnant following the Immaculate Conception whereas Christina had never been pregnant. After finally being seized and tortured, her breasts began to flow with the clearest oil which she used for flavoring her meal and as an ointment for her festering wounded limbs (17). Margaretha Ebner, a Dominican nun who lived during the mid-1300’s began to miraculously lactate after a small figure of the Christ-child asked her to be nursed (18). Other mystics have experienced non-puerperal lactation when envisioning the baby Jesus, including Lidwina of Schiedam, Gertrude of Delft, and Veronica Giuliana (19).

Various folkloristic methods for inducing lactation in older literature are using plant poultices covered with heated stones over the breasts of a virgin, and infusion and massage of plant ash into breast incisions to promote relactation (11). Finally, the Hebrew Talmud features an interesting anecdote of a nursing woman who died suddenly. Surprisingly, her widowed husband gained the ability to lactate his newborn (Bab. Talmud, Shabbat, fol. 53b). Similar cases were reported by Aristotle and others.

### Galactorrhea and hyperprolactinemia

The term galactorrhea denotes breast secretions in non-puerperal women and in many times implies a disease state (20). Whereas up to a quarter of normal women who have been pregnant in the past may have small amounts of breast secretions that lack any clinical significance, no breast secretion whatsoever are detectable in normal menstruating nulligravid women. Since PRL is mandatory for milk production, aberrent physiology may be assumed as the cause of galactorrhea. Apart from stimulating milk production, elevated PRL levels (termed hyperprolactinemia) may affect fertility. PRL exerts a negative feedback on pituitary hormones and causes post-partum amenorrhea. Teleologically, it has been hypothesized that this effect of PRL prevents consecutive pregnancies, thereby promoting prolonged breast-feeding.

Galactorrhea is usually the result of either a failure of the hypothalamic inhibition of PRL secretion or autonomous PRL secretion (21). Loss of inhibition may be the result of trauma, damage to the pituitary during surgery or due to diseases of the central nervous system such as acromegaly, carniopharyngioma and granulomatous diseases of the hypothalamus, hypothyroidism and chronic renal failure. Drugs that cause hyperprolactinemia include metoclopramide, phenothiazines, antidepressants, risperidone, verapamil and methyldopa (22). Increased secretion usually results from autonomous production by pituitary tumors (prolactinomas) and rarely, PRL-secreting tumors of the lung or kidneys (23).

The presence of galactorrhea mandates a clinical investigation in order to establish its cause. First, adequate medical history, physical examination and routine laboratory testing should be employed to ascertain the presence of galactorrhea and document hyperprolactinemia by measuring serum PRL level. If hyperprolactinemia is present, drugs and systemic diseases should be ruled out (22). After other causes have been ruled out, MRI of the brain is performed in order to look for prolactinoma (microadenoma if <10mm or macroadenoma if 10mm or more).

Treatment options for hyperprolactinemia include correction of underlying causes or suppression of PRL secretion in the case of a secreting tumor. Suppression of PRL secretion may be achieved by drug therapy with dopamine agonists (bromocriptine, cabergoline, pergolide), surgery (usually by a transsphenoidal approach) and to a lesser extent radiotherapy. The choice of the treatment modality depends on the size of the tumor, its location, the presence of infertility and/or hypogonadism and family planning and algorithms have been proposed (22, 24).

Therefore, hyperprolactinemia causes galactorrhea. Although the nutritional value of produced milk and its volume are poorer than with normal lactation, it is possible that galactorrhea has been viewed within developing communities as a supernatural ability to breast-feed without pregnancy. The association of infertility with galactorrhea fits quite well with the lore of the Bedouin tribes of the Negev.

### Induced Lactation

Induced lactation is the process by which a non-puerperal woman is stimulated to lactate. From the earliest times, including the time of Hippocrates, it has been thought that lactation may occur in virgins (25). It is therefore not a new concept and it is widespread among many cultures. Historically, induced lactation has commenced after an infant’s mother has died during labor or puerperium or was unable to breast-feed for some reason. Breast-feeding was usually induced in grandmothers, a procedure termed relactation (stimulation of breast-feeding in a woman who has given birth in the past) but also in nulligravid women (26).

Most documented cases of induced lactation began to appear in the medical literature early in the 20^th^ century. This practice has been encountered in New Guinea (27), Zulu tribes in Africa (28), Yoruba community in Nigeria (29), Aboriginal communities in Australia, and in Tierra del Fuego, South America. The techniques employed by most of these cultures are suckling, the application of heat and massage of the breast, and drinking coconut milk (25). In addition to the anthropological evidence, scientific data has also begun to accumulate in the 20^th^ century with regard to the physiologic basis of induced lactation. Induced lactation has been observed and also induced experimentally in dogs, goats, and rats (30), and has even been documented in adult men (31).

Until recently, the medical literature has reported this phenomenon anecdotally or as a part of the discussion of aberrant lactation. Today, this issue has gained much interest in the modern and industrialized world owing to the wish of many adopting mothers to nurture adopted infants. In the case of surrogate mothers, preparations for induced lactation begin even before birth.

Lactation may be induced by various methods. Frequent daily sessions of manual or mechanical nipple stimulation should be carried out and the woman should be properly educated about the concept of induced lactation, especially if she has not breast-fed previously. Protein-rich dietary supplementation should be provided. The administration of drugs that promote milk production (galactogogues) such as estrogens or chlorpromazine, and the administration of the hormone oxytocin, a hormone that activates the milk let-down reflex, should be strongly considered (31).

In unprivileged countries induced lactation is used as an emergency measure, especially by African communities, for preventing neonatal death when certain women fail to breast-feed (32). In such cases, no pre-partum preparations are possible, but current experience suggests a high success rate in these settings, probably owing to the many years of traditional experience with induced lactation.

## CONCLUSIONS

This paper has reviewed the practice of breast-feeding and wet-nursing in Islam, an issue that has received much of attention in Islamic societies both socially and religiously, and constitutes an important theme in Islamic folklore. While breast-feeding has intentionally assumed cultural and religious roles on numerous occasions, such as in the case of Islam, various unexplained phenomena have led to the emergence of the rich folklore and myth surrounding it.

The case of being able to breast-feed without pregnancy (non-puerperal lactation) is intriguing and has been documented in various societies throughout centuries. Nevertheless, it serves as an excellent example for the value of utilizing contemporary scientific knowledge in order to elucidate the origin, anthropology and evolvement of ancient myth and superstition.
